# A novel method for measuring pulmonary artery pressure by high-frequency ultrasound-guided transthoracic puncture in rats

**DOI:** 10.3389/fcvm.2022.995728

**Published:** 2022-09-28

**Authors:** Xiaofeng Zhang, Jingtao Li, Decai Zeng, Chunting Liang, Yanfen Zhong, Tongtong Huang, Yingying Mo, Huaqing Rao, Xiaoxiong Pan, Ji Wu

**Affiliations:** Department of Ultrasonic Medicine, First Affiliated Hospital of Guangxi Medical University, Nanning, China

**Keywords:** ultrasound guidance, pulmonary hypertension, rat model, pressure measuring method, novel method

## Abstract

**Objectives:**

The success of the rat model of pulmonary hypertension (PH) is primarily dependent on the measurement of pulmonary artery pressure. We herein demonstrate a novel method for measuring pulmonary artery pressure through a high-frequency ultrasound-guided transthoracic puncture in rats. The efficacy and time of this novel method are also discussed.

**Methods:**

A single subcutaneous injection of monocrotaline (MCT) was used to establish a rat model of PH. Through the heat shaping method, the tip of that puncture cannula was maintained at a certain angle after the needle core was removed. In-plane real-time guided trocar puncture of the right ventricular outflow tract was performed in the short-axis section of the parasternal aorta. The external pressure sensor was used to record the real-time waveform of right ventricular systolic pressure, pulmonary artery systolic pressure, and diastolic pressure.

**Results:**

The success rates of which using this novel method in the model group and the control group were 88.5 and 86.7%, respectively. The time of puncture pressure measurement was 164 ± 31 and 235 ± 50 s, respectively. The right ventricular systolic blood pressure, pulmonary systolic blood pressure, and diastolic blood pressure of the model group were higher than those of the control group.

**Conclusion:**

The modified method for trocar is helpful for accurately positioning pulmonary artery manometry. The method described in this paper has a high success rate and short operation time. It can simultaneously measure systolic blood pressure, diastolic blood pressure, and mean pressure of the right ventricle and pulmonary artery. It has a broad application prospect in verifying the rat PH model and pulmonary artery pressure monitoring.

## Introduction

Pulmonary hypertension (PH) is a fatal disease with progressive aggravation, mainly characterized by pulmonary vascular remodeling and progressive pulmonary vascular resistance, leading to right heart failure and death (1). Accurate measurement of pulmonary artery pressure is the critical technology to evaluate the successful preparation of the pulmonary arterial hypertension model. Traditional pulmonary artery pressure measurement adopts the right heart catheter method. The catheter passes through the peripheral vein-right atrium-right ventricle-pulmonary artery and needs to undergo many physiological bends. The catheter is easy to pierce the vein and heart, causing hemorrhage or death and failure of pressure measurement in rats (2).

Animal models play an irreplaceable role in revealing pulmonary vascular remodeling and its pathophysiological mechanism. Hence, it is necessary to consider the cost and time of establishing a PH model in SD rats. Experimental animal ethics requires the three principles of substitution, reduction, and optimization in medical research to ensure the welfare of experimental animals. Researchers urgently need a better pulmonary artery manometry to improve the success rate and reduce animals’ trauma and unnecessary death (3).

In recent years, ultrasound-guided puncture technology had made significant progress in the field of diagnosis and treatment, and it was one of the most accurate, safe, and efficient diagnosis and treatment schemes (4–6). With the application of high-resolution ultrasound in small animal imaging, the puncture area could be accurately identified and located (7–9). In this study, the needle tip and catheter were accurately located in the right ventricular outflow tract and pulmonary artery by high-frequency ultrasound, which verified the feasibility of pulmonary artery manometry.

## Materials and methods

### Establishment and grouping of experimental mode

All experiments were conducted following the Guide for the Care and Use of Laboratory Animals and authorized by the Institutional Animal Care and Use Ethics Committee (Animal License No. SYXK GUI 2020-0004). Sixty adult Sprague-Dawley rats were randomly divided into the blank control group and PH group, each with 30 rats. The PH group established the rat PH model according to Bueno-Beti et al. (10), a single subcutaneous injection of 1 ml of monocrotaline (MCT) (60 mg/ml; Sigma, St. Louis, MO., USA). Then, rats were raised for 4 weeks to induce PH. Rats in the control group were injected with isotonic saline (PH7.2) in the same way. In this study, we used 8-week-old male SD rats weighing 220–270 g.

### Experimental consumables and primary instruments

Experimental materials such as pentobarbital sodium (40 mg/kg), heparinized sterile normal saline (100 U/ml), 75% ethanol, sterile ultrasonic coupling agent, vascular clamp, and syringe were provided by the First Affiliated Hospital of Guangxi Medical University. Other instruments included 20G disposable intravenous indwelling needle (B. Braun Medical Ltd, Sheffield, UK), BL-420 Biological Function Experimental System (Chengdu Taimeng Software Co., Ltd.), GE Vivid E95 ultrasonic diagnostic instrument (general electric co., Fairfield, CT, USA).

### Anesthesia and instrument preparation

After weighing, all rats were injected with pentobarbital sodium (40 mg/kg) intraperitoneally. The depth of anesthesia was checked by pinching the hind toes. Then the hair on the chest of rats was removed. The rats were placed in the left lateral position, and the room temperature was monitored to ensure the proper environmental temperature. Eye ointment was used to prevent dryness under anesthesia. An experienced ultrasound interventional doctor completed the puncture operation.

**FIGURE 1:**
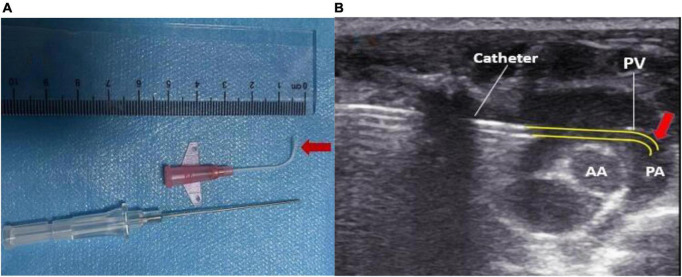
**(A)** The cannula tip was maintained at a bend of 90° by 1 cm using heat shaping. **(B)** Pressure measurement over the pulmonary valve reached by the reshaped catheter. PA, pulmonary artery; AA, aorta; PV, pulmonary valve.

The tip of the intravenous indwelling needle cannula was thermoformed 1 cm above the external flame of the alcohol lamp to maintain a 90° bend angle (Figure 1A). The tip of the needle was maintained at a certain angle after the needle core was pulled out and smoothly passed through the pulmonary valve (Figure 1B).

Connecting the tee pipe with the pressure transducer and 20G puncture needle for later use, the assistant filled the transducer, catheter, and puncture needle with heparinized sterile saline (100 U/ml) to ensure no bubbles in the transducer and catheter. The syringe barrel was held on that tee pipe to maintain the close pressure. At the same time, it was convenient to flush the catheter with heparin saline for anticoagulation.

### Echocardiographic evaluation

GE Vivid E95 ultrasonic diagnostic instrument was equipped with an L8-18i transducer high-frequency superficial probe, with the highest frequency of 18 MHz. The diastolic transverse diameters of the right ventricle and right atrium were measured in an apical four-chamber view. The anteroposterior diameter of the right ventricular outflow tract and the width of the pulmonary artery were measured on the parasternal short-axis view. Rats with congenital stenosis of the right ventricular outflow tract and pulmonary artery (inner diameter less than 2 mm) were excluded.

### Puncture method

**FIGURE 2:**
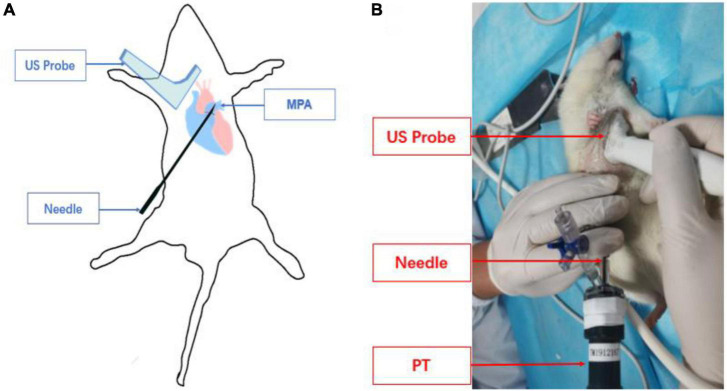
Schematic diagram of pulmonary artery puncture guided by high-frequency ultrasound. **(A)** Schematic diagram of needle track and transducer positioning. **(B)** The actual operation of pulmonary artery puncture is guided by ultrasound.

The operator routinely wore sterile gloves and applied sterile coupling agents evenly on the probe. The probe was held by the right hand to make it perpendicular to the skin. The pulmonary artery and its surrounding tissue structure were explored to determine the puncture position and puncture angle. The assistant lifted the skin with hemostatic forceps at the puncture point to facilitate needle insertion. Operators used the in-plane method to puncture the right ventricular outflow tract in the parasternal PA short-axis view (Figure 2A). Ultrasound guided the needle tip to enter the chest cavity through the fourth intercostal space at the right edge of the sternum in real-time (Figure 2B). The needle insertion direction in the thymus behind the sternum was adjusted according to the parasternal PA short-axis view. When the needle tip entered the right ventricular outflow tract on the ultrasound image, the operator observed whether the puncture needle returned blood, or took 0.5 ml of rat blood for pellet injection and verified it on the ultrasound image.

**FIGURE 3:**
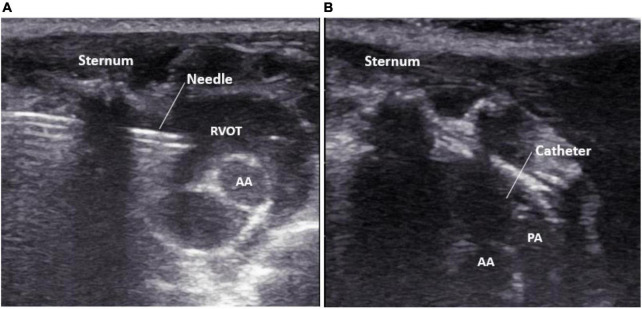
Real-time display of needle tip guided by high-frequency ultrasound. **(A)** The puncture needle tip is located in the right ventricular outflow tract; **(B)** the catheter tip is located in the pulmonary artery. PA, pulmonary artery; AA, aorta; RVOT, right ventricular outflow tract.

Meanwhile, the assistant turned on the pressure transducer to observe the right ventricular pressure curve in real-time. When the needle tip entered the right ventricular outflow tract, the right ventricular waveform changes were displayed on the pressure transducer. After recording a right ventricular pressure curve, the needle core was withdrawn, and the catheter was sent into the pulmonary artery along the direction of the pulmonary artery under the guidance of ultrasound in real-time, and the pulmonary artery pressure curve was recorded (Figures 3A,B). A video on ultrasound-guided percutaneous transthoracic pulmonary artery pressure measurement is shown in Supplementary Video 1.

### Operation time measurement

Start timing from the appropriate puncture point was selected by the probe. When the depth of the needle was about 18 mm, it would reach the right ventricular outflow tract. When continued to advance for about 10 mm, we observed the stable pulmonary artery pressure waveform, which would be regarded as a successful operation, and then the timing was stopped. Suppose it was shown that the needle tip was in the pulmonary artery, no pulmonary artery waveform appeared during the process. In that case, the puncture needle could be retracted slightly and then rotated into the needle to try until a stable pulmonary artery waveform appears. When a rat died, or a large amount of pericardial effusion occurred during the operation, it was regarded as operation failure. The time for successful puncture pressure measurement of each rat was recorded, and the success rate was calculated.

### Pathological examination

The rats were killed after the puncture. The left and right pulmonary lobes were examined for acupuncture injury by the naked eye. Meanwhile, rats’ upper left lobe tissues in the experimental and control groups were taken. The left main bronchus was perfused with 4% paraformaldehyde, and the rat’s left upper pulmonary lobe tissue was taken. The paraffin-embedded sections (5 μm) were fixed with paraformaldehyde for 48 h and then stained with hematoxylin and eosin (H&E). The sections were observed under the Nikon 80i microscope (Nikon, Japan).

Ten fields of view were randomly selected from the lung sample of each rat, and the arterioles less than 50 μm in diameter were quantitatively measured. The total vascular area (TA) and lumen area (LA) of pulmonary arterioles were measured by IPP 6.0 image analysis software. The percentage of wall area (WA) to the total area of vessels [WA%, WA% = (TA–LA)/TA × 100%] was calculated.

### Statistical analysis

All statistical analyses were performed using commercially available software (SPSS, release 19.0). Data were expressed as means ± SD. An independent sample *t*-test was used for comparison between the two groups. Categorical data were compared by Pearson’s Chi-square test. A *P*-value of < 0.05 indicated a statistically significant difference.

## Results

### General situation of rats

The rats successfully modeled by MCT in the experimental group had dark hair color, cyanosis of oral mucosa and extremities, shortness of breath, decreased food intake, and weight loss. In contrast, the control group had no abnormal performance. There were 30 rats in the control group and 30 rats in the PH group. The right atrium transverse diameter (RATd), right ventricular diameter (RVTd), pulmonary artery diameter (PAd), and thickness of the right ventricular anterior wall (RVAW) in the MCT group were higher than those in the control group (Table 1).

**TABLE 1:** The detailed parameters of pulmonary artery pressure were measured by puncture in the control group and the monocrotaline (MCT) group.

	Control group	MCT group	*p*
n	26	23	
Weight (g)	372.55 ± 11.97	273.95 ± 11.89*	<0.0001
HR (bpm)	345.0 ± 7.7	339.7 ± 8.7*	0.036
RVOTd (mm)	2.84 ± 0.18	3.68 ± 0.27*	<0.0001
RVTd (mm)	3.38 ± 0.23	4.87 ± 0.50*	<0.0001
RATd (mm)	3.45 ± 0.22	5.10 ± 0.42*	<0.0001
PAd (mm)	2.72 ± 0.10	3.67 ± 0.10*	<0.0001
RVAW (mm)	0.75 ± 0.05	1.45 ± 0.12*	<0.0001
Puncture time(s)	235 ± 50	164 ± 31*	<0.0001

HR, heart rate; RVOT, right ventricular outflow tract; RVTd, right ventricular diameter; RATd, right atrium transverse diameter; Pad, pulmonary artery diameter; RVAW, Right ventricular anterior wall thickness. *P < 0.05 vs. control group by the independent sample *t*-test.

### Ultrasound-guided puncture

We performed ultrasound-guided transthoracic puncture of the pulmonary artery in 60 adult male SD rats and 49 of them were successfully tested. In the MCT group, four rats died only due to anesthesia accidents, three rats experienced cardiac tamponade due to puncture accidents, and 23 rats were successfully punctured and measured. In the control group, 26 rats were successfully punctured, and four rats suffered from pericardial tamponade. The puncture success rates of the two groups were 88.5 and 86.7%, respectively. The overall success rate of transthoracic pulmonary artery pressure measurement guided by high-frequency ultrasound was 87.5%. According to the Chi-square test results of continuous correction, there was no significant difference in the success rate of puncture between the two groups (*P* = 1). The time required for the successful measurement of pulmonary artery pressure in the MCT and control groups is shown in Table 1. The time needed for puncture pressure measurement in the two groups was statistically significant (235 ± 50 s, *n* = 26 vs. 164 ± 31 s, *n* = 23, respectively, *p* < 0.01).

### Remodeling and stenosis of pulmonary muscular arterioles

**FIGURE 4:**
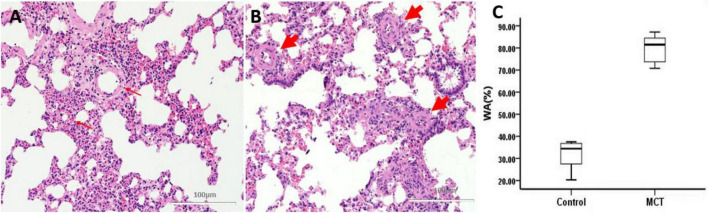
Pulmonary muscular arterioles remodeling and stenosis. **(A)** Control group, normal rat pulmonary arterioles (*fine arrow*); **(B)** MCT group, pulmonary arteriole remodeling (*thick arrow*). Bar = 100 μm. **(C)** The percentage of wall area to the total area of vessels (WA%). The thick arrow shows the muscular pulmonary arterioles.

Macroscopic examination of the left and right pulmonary lobes showed that no acupuncture injury or bleeding point was found in all rats. Pulmonary vascular remodeling was induced by MCT in SD rats. HE staining showed that the control group’s pulmonary artery wall and structure were intact without interstitial inflammatory cells (Figure 4A). Inflammatory cells, alveolar damage, and pulmonary interstitial hyperplasia were found in the interstitial tissue of the MCT group (Figure 4B). Compared with the control group, the WA% of the pulmonary artery in the MCT group increased significantly (Figure 4C).

### Pressure and waveform

The measured values of right ventricular systolic pressure, pulmonary artery systolic pressure, and diastolic pressure in the MCT group were higher than those in the control group (*P* < 0.01), and the difference was statistically significant. The pressure values obtained by ultrasound-guided pulmonary artery puncture pressure measurement could distinguish the PH group from the control group and simultaneously measured the right ventricular systolic blood pressure, pulmonary artery systolic blood pressure, and diastolic blood pressure in rats (Table 2).

**TABLE 2:** Comparison of pressure measurements between MCT group and control group.

Group	RVP		PAP	
		
	RVSP (mmHg)	PASP (mmHg)	PADP (mmHg)	PAMP (mmHg)
Experimental	41.6 ± 11.8	41.5 ± 10.9	22.3 ± 4.4	28.7 ± 6.6
Control	22.0 ± 4.3	22.0 ± 4.1	6.1 ± 2.1	11.4 ± 2.8
P	<0.0001	<0.0001	<0.0001	<0.0001

RVP, right ventricular pressure; RVSP, right ventricular systolic pressure; PAP, pulmonary artery pressure; PASP, pulmonary artery systolic pressure; PADP, pulmonary artery diastolic pressure; PAMP, pulmonary arterial mean pressure.

**FIGURE 5:**
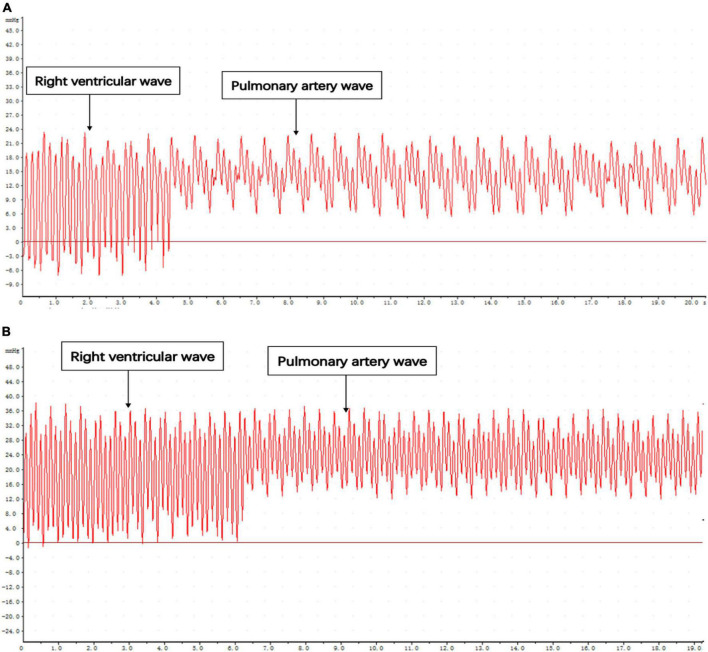
Pressure curve of catheter entering right ventricular outflow tract and pulmonary artery. **(A)** Control. **(B)** MCT group.

When the catheter was located in the right ventricular outflow tract, the right ventricular wave suddenly rose and dropped. The diastolic pressure fluctuates around 0, and the difference between systolic and diastolic pressure is significant. When the catheter was inserted through the pulmonary valve for about 0.5 cm, the amplitude decreased, and the systolic pressure of the pulmonary artery was slightly smaller than that of the right ventricle. The diastolic blood pressure increases and a deep descending wave will appear every four waves under the influence of respiration (Figures 5A,B).

## Discussion

PH caused by various causes has always been one of the hot spots in the cardiovascular research field. The measurement of PH is the key to verifying the success of the PH model. It is also an important indicator for evaluating various drugs’ efficacy in treating PH and therapeutic strategies. The traditional right heart catheter method was widely used in measuring pulmonary artery pressure in humans and large animals (11, 12). At present, there is no pressure measuring catheter for rats and small animals. According to the anatomical structure of rats, researchers made the right heart catheter with a polyethylene tube. They baked the corresponding curved catheter with the external flame of the alcohol lamp until the correct pulmonary artery waveform appeared (13). This method belongs to the blind insertion method, and its skills are complex, so it takes a long time to train to obtain a higher success rate.

Tontodonati et al. (14) used a Millar pressure sensor catheter to enter the right ventricle of rats through the right external jugular vein approach and take pressure measurements. The experimenter indirectly identified and corrected the position of the catheter tip through pressure signals, which reduced the operation time compared with the self-made catheter. However, the subminiature catheter pressure sensor and its supporting equipment used in this technology are expensive and vulnerable parts, which may have excellent pressure measurement time variability due to the standard anatomical variation of the jugular vein. Because of the small size of rats, the pressure measuring catheter only reaches the right ventricle and measures the right ventricular pressure. Severe PH causes right ventricular failure, which leads to high mortality in rats (15). In rats with severe PH, abnormal conduction of the right ventricle and unstable depolarization of the myocardium might induce fatal arrhythmia. Still, the right cardiac catheter *via* peripheral vein approach could aggravate this arrhythmia and lead to the failure of manometry (16).

In recent years, ultrasound-guided minimally invasive diagnosis and treatment technology had been widely used. Uzumcugi et al. (17) used ultrasound-guided puncture to set the right internal jugular vein in low-weight infants. The first puncture success rate was 70%, and the overall success rate was 88%. The inner diameter of the pulmonary artery in rats is about 4 mm, and its inner diameter and depth from the skin are similar to those in the above puncture target area. The literature had reported (18) that ultrasound-guided percutaneous transhepatic biliary drainage can puncture a 2 mm non-dilated bile duct. Its puncture path is long, and its puncture difficulty is even more severe than that of an ultrasound-guided puncture of a pulmonary artery. Its puncture success rate is 94.3%, which provides a theoretical basis for reference for this experiment.

In this study, the right ventricular outflow tract was punctured through the chest wall, which significantly shortened the catheter pressure measurement path and guided the pulmonary artery pressure measurement by ultrasound visualization in real-time. This method did not need mechanical ventilation, short operation time, and was less invasive than the thoracotomy approach. Animals can be in a stable state for a long time without tracheal intubation and ventilator-assisted ventilation and maintain normal intrathoracic negative pressure, thus ensuring steady right heart filling pressure and pulmonary artery pressure (19). In this study, seven rats failed in puncture and pressure measurement. The autopsy confirmed that multiple improper punctures resulted in multiple myocardial injuries. Since the puncture technique was not sufficiently skilled at the initial stage of the experiment, repeated punctures resulted in cardiac tamponade.

The external flame reshaping method with an alcohol lamp successfully maintained the tip of the cannula at a certain angle. Thus, the tip of the cannula could smoothly pass through the pulmonary valve without piercing the pulmonary artery, which will ensure the stability of the pressure measurement process. Transthoracic pulmonary artery pressure measurement of SD rats under ultrasound guidance could display in real-time that the puncture needle was located in the right ventricular outflow tract. Then, the cannula was pushed into the pulmonary artery under ultrasound guidance for pressure measurement, which could simultaneously measure right ventricular and pulmonary artery pressure. Their systolic pressure was approximately equal. Some scholars used right ventricular pressure to indirectly reflect the changes in pulmonary artery pressure (14, 20), but their diastolic pressure and mean pressure were pretty different. According to the European Guidelines for Diagnosis and Treatment of PH (21), the diagnosis of PH was based on the mean pressure of the pulmonary artery measured by right cardiac catheterization. Therefore, it was more clinically significant to measure systolic pressure, diastolic pressure, and mean pressure of pulmonary artery simultaneously by transthoracic puncture of pulmonary artery guided by high-frequency ultrasound. The waveform and pressure value of pulmonary artery pressure measured in this study were consistent with the results reported in previous literature (10, 22). At the same time, this study also verified the feasibility of puncturing the right ventricular outflow tract through the chest wall under ultrasound guidance, which could provide a new interventional path for minimally invasive cardiac surgery treatment.

## Conclusion

This study showed that transthoracic pulmonary artery puncture under the guidance of high-frequency ultrasound can achieve visual pulmonary artery pressure measurement, and the improved trocar method contributes to the accurate positioning of pulmonary artery pressure measurement. The method is time-saving and accurate and can successfully measure the pulmonary artery systolic blood pressure, diastolic blood pressure, and mean pressure. It has a high application prospect in verifying the rat PH model and monitoring pulmonary artery pressure.

## Data availability statement

The original contributions presented in this study are included in the article/Supplementary material, further inquiries can be directed to the corresponding author.

## Ethics statement

This animal study was reviewed and approved by the Guangxi Medical University. Written informed consent was obtained from the owners for the participation of their animals in this study.

## Author contributions

XZ and JW conceived the study. XZ, JL, DZ, and CL performed the experiments. XZ, XP, and JL analyzed the data. XZ, YM, HR, and YZ interpreted the results of the experiments and drafted the manuscript. JW designed the research. All authors contributed to the article and approved the submitted version.

## Supplementary material

Supplementary Video Practical demonstration of ultrasound-guided percutaneous transthoracic pulmonary artery pressure measurement.
